# Self- and parent-reported executive function and self-determination in autistic teens: domain-specific relationships and cross-rater considerations

**DOI:** 10.1186/s11689-026-09703-2

**Published:** 2026-08-03

**Authors:** Jennifer R. Bertollo, Lauren Baczewski, Hannah Behar, Elizabeth R. Black, Tara Anderson, K. Sharp, Andrea Lopez, Chloe C. Neuman, Alyssa Verbalis, Cara E. Pugliese, Lauren Kenworthy

**Affiliations:** 1 Children’s National Hospital, Washington, DC US; 2 Curry College, Milton, MA US; 3 School of Education, Indiana University Bloomington, Bloomington, IN US; 4 Loyola University Maryland, Baltimore, MD US

**Keywords:** Autism, Executive Function, Self-Determination Opportunities, Self-Determination Capacity, Self-Report, Adolescence, Young Adulthood

## Abstract

**Background:**

Self-determination, or the degree to which an individual perceives they have “causal agency” over their life plans, is an important predictor of quality of life, employment, and education for autistic people. However, little research has explored predictors of self-determination in autism. Limited research links executive function (EF) to self-determination, but tends to focus on EF globally and fails to address specific EF subdomains. Report of cross-rater links between EF and self-determination (e.g., parent-report EF and self-report self-determination) is also missing. As such, the current study evaluates the relation between real-world EF subdomains and self-determination, both within and between raters.

**Methods:**

226 autistic teens ages 14–22 years (*M* = 16.17; 73% male, 75% white, 89% non-Hispanic) and their parent/caregiver completed rating scales about the teens’ EF and self-determination at enrollment for one of two EF intervention studies. Through a series of linear regressions, the relationship between EF subscales on the BRIEF-2 and Self-Determination capacity and opportunity at home (on the AIR Self-Determination Scale) were evaluated within each rater (self or parent). Exploratory analyses repeated these models across rater.

**Results:**

Within self-report, greater EF challenges with flexibility and planning predicted lower capacity for self-determination. Within parent-report, relationships with self-determination capacity were generalized across EF subdomains (e.g., self-monitoring, flexibility, initiation, working memory, planning/organizing). EF subdomains such as flexibility, planning, working memory, and emotion regulation demonstrated cross-rater relationships with self-determination. Skills such as organization of materials and inhibition appeared to be uniquely important for understanding opportunities provided for teens to be self-determined at home.

**Conclusions:**

The current study replicates and extends prior findings linking EF to self-determination in autism, by highlighting possible EF subdomains that relate to self-determination capacity and opportunity, and demonstrating that some of these relations are robust enough to be seen across teen and parent raters. Findings suggest that supporting specific EF skills such as flexibility, planning, initiation, inhibition, and emotional control may be important for facilitating self-determination. Findings also underscore the importance of providing opportunities for teens to play a central role in developing and executing plans toward their goals.

**Clinical trial registration:**

#NCT05131659, #NCT05017779.

## Background

### Self-determination in autism

Self-determination is an important predictor of quality of life, employment, and education for people with developmental disabilities, including autism (Martino et al., 2025). Self-determination refers to the degree to which an individual feels that they have “causal agency” over their life goals, choices, and plans [1, 2]. Becoming an agent in one’s own life requires both having the capacity to take action toward goals and being given sufficient opportunities to do so. As described by Wehmeyer and colleagues [2], self-determination includes four key characteristics: self-regulation, psychological empowerment, autonomous functioning, and self-realization. Autistic people tend to have lower self-determination than their non-autistic peers [3], which is thought to be due to the relative lack of opportunities they are given to develop and/or practice key skills, such as autonomously determining their supports, care, and goals [4–6]. Further, societal stigma drives assumptions about the capacity of autistic people to be agents in their own lives, and these assumptions often result in fewer opportunities to be self-determined [7].

Opportunities for self-determination are especially critical during the transition to adulthood, which includes future planning that is grounded in an individuals’ personal values and goals [8]. Society often holds traditional views and assumptions of what “successful” adulthood should look like and autistic young adults have preferences, values, and goals for themselves that may align with or differ from these expectations [9]. Therefore, it is crucial that they are empowered to make decisions about their own futures in a way that prioritizes their autonomy [4, 7].

Budding research exploring self-determination in autism has shown that self-determination is associated with a host of positive outcomes for autistic people, including greater self-reported quality of life [10, 11] and the pursuit of postsecondary education and employment [7]. However, much of the early literature on this topic centered solely on the accounts of parents/caregivers or healthcare providers, lacking the first-hand perspectives of autistic individuals on their own self-determination experiences [10]. Previous research on constructs such as anxiety and executive function have demonstrated that autistic individuals offer valuable insight into their internal experience that is not captured from their parents’ report alone [12, 13]. Additionally, there are often significant discrepancies between self and parent ratings in many domains, such as daily living skills, quality of life, unmet service needs, and even self-determination [14, 15]. As such, prior research emphasizes the need for multiple reporters in assessing predictive outcomes, and incorporating the individual’s self-report, especially when assessing internal experiences [14, 16]. Further, collecting data from multiple reporters allows for comparison across raters, which is critical for understanding multi-dimensional behavioral and mental health related experiences [16].

### Predictors of self-determination in autism: the role of executive function

While the importance of self-determination to adulthood outcomes in autism is clear, less is known about factors that predict or relate to self-determination. Evidence suggests that executive function (EF) skills (e.g., cognitive and behavioral flexibility, planning, emotion regulation, working memory) may play an important role in self-determination. As described by Shogren and colleagues [17], self-determination requires successful “self-regulation and goal-directed action,” which rely on core EF skills. In theoretical models of self-determination, cognitive flexibility and inhibitory control are critical to taking agentic action, as individuals need to inhibit distractions and act flexibly to navigate barriers in pursuit of their goal [1, 17]. Autistic individuals often experience challenges with EF [18, 19] and benefit from targeted intervention to build EF skills [20]. In the context of these relative challenges, studies have begun to examine the link between EF skills and self-determination in autism.

Among autistic adolescents and young adults without intellectual disability, fewer global (or overall) EF challenges are related to greater capacity for self-determination [21]. Tomaszewski and colleagues [21] measured global EF skills using the Behavior Rating Inventory of Executive Function (BRIEF-2 and BRIEF-A) [22, 23], which provides a Global Executive Composite (GEC) score that sums items across EF subdomains. Autistic adolescents reported on their EF and self-determination, and parents reported on their child’s EF and self-determination skills, making their study one of the few on this topic to obtain both self- and parent-report. However, only within-rater relationships are reported in results, wherein self-reported global EF predicted self-reported self-determination and parent-reported global EF predicted parent-reported self-determination. Only one study to our knowledge has examined the relationship between EF subdomains and self-determination, using computerized task-based measures of EF skills (i.e., Matrix Span, Stroop, Flanker, and Tower of Hanoi tasks [24]). In this sample of young people with intellectual disability (approximately half of whom were also diagnosed with autism), Lui and colleagues [24] found that self-rated self-determination capacity was related to planning-related EF skills, as planning mediated the relationship between self-determination and quality of life. To our knowledge, no studies have examined subdomains of informant-reported, real-world EF skills as predictors of self-determination among autistic young people without intellectual disability, or cross-rater relationships in these domains.

### Current study

To address these gaps in prior literature, the current study aims to explore: (1) whether there are significant relationships between subdomains of informant-reported EF and self-determination, and (2) whether these relationships can be seen across reporters (e.g., self-reported EF to parent-reported self-determination). In a large sample of autistic adolescents, we evaluate these aims through parent- and self-report rating scales of autistic teens’ EF and self-determination. Given the limited literature on self-determination and EF in autism, we assessed relationships between all subdomains of EF and self-determination in the current study. However, based on the critical role of cognitive flexibility and inhibitory control in theoretical models of self-determination [17], we hypothesized that EF subdomains on the BRIEF-2 that measure flexibility (Shift scale) and inhibitory control (Inhibit scale) would be significant predictors of concurrent self-determination. Additionally, given prior self-determination work in autism suggesting relatively low opportunities for planning and monitoring plans in the home environment [15], we hypothesized that planning-related EF skills (e.g., Plan/Organize on the BRIEF-2) would emerge as an additional predictor of self-determination. Finally, we hypothesized that some of these EF subdomains would predict variance in self-determination across raters, with self-reported EF domains predicting parent-reported self-determination, and vice versa.

## Methods

### Participants

Participants included 226 autistic teens between the ages of 14.08 and 22.15 years (*M* = 16.17, *SD* = 1.34; see Table 1 for complete demographics). In the current study, we use the word “teens” throughout to refer to high school students who are 14 through 22 years of age. Participants were primarily white (75%), non-Hispanic (89%) males (73%). For each teen, one parent/caregiver (*n* = 226; 76% mothers) also participated and completed measures about their teen. The current study includes baseline data from teens who were enrolled in one of two EF intervention trials: one in a hospital research laboratory setting (*n* = 64) and one in high schools (*n* = 162). Baseline measures completed at the time of enrollment in these treatment trials were analyzed in the current paper.

Recruitment materials for families described the intervention as a group-based curriculum that “helps prepare high school students for post-secondary education through learning to set goals, being flexible, planning, self-advocating, increasing motivation, and getting work done on their own.” Eligibility criteria for the two larger studies were as follows. For the laboratory-based study, participants needed to be 14–18 years of age at enrollment and have a full-scale IQ (FSIQ) ≥ 80, either by report within the past two years, or on the Wechsler Abbreviated Scales of Intelligent, 2nd Edition [25] in current study. Participants had to meet DSM-5 criteria for a diagnosis of autism spectrum disorder, as evidenced by a prior autism diagnosis or classification, confirmed by the Social Communication Questionnaire (SCQ [26]), Autism-Diagnostic Observation Schedule, Second Edition (ADOS-2 [27]) and/or clinical judgment of a licensed psychologist on the research staff. Although the current study recruited autistic individuals, if a participant referred for the current study with suspected autism but without a formal prior diagnosis (*n* = 2), they were diagnosed through the current study, including a review of intake materials, SCQ, ADOS-2, and expert judgment of the clinical psychologist. This study also included neuroimaging, so participants were excluded if they had any significant neurological disorders aside from autism or attention-deficit/hyperactivity disorder (ADHD; e.g., epilepsy, hydrocephalus, central nervous system infection, stroke) or any contraindications for neuroimaging (e.g., metal implants, dental braces, pregnancy).

**Table 1:** Participant demographics

	School-Based Study (*n* = 162)		Lab-Based Study *(n* = 64)		Overall Sample *(N* = 226)	
	M (SD)	Range	M (SD)	Range	M (SD)	Range
Participant Age (years)	16.25 (1.38)	14.14–22.15	15.94 (1.20)	14.08–18.15	16.17 (1.34)	14.08–22.15
Full-Scale IQ (Standard Score)^a^	98.06 (16.44)	61–135	109.66 (13.13)	83–142	101.36 (16.40)	61–142
Autism Characteristics (SRS-2 Total T-Score)^b^	71.77 (11.76)	42–98	73.28 (9.91)	50–99	72.23 (11.22)	42–99
	***n***	**%**	***n***	**%**	***n***	**%**
Gender						
Female	33	20.37%	14	21.88%	47	20.80%
Male	121	74.69%	45	70.31%	166	73.45%
Non-Binary	7	4.32%	5	7.81%	12	5.31%
Not Specified/Prefer Not to Answer	1	0.62%	0	0%	1	0.44%
Race^c^						
American Indian/Native American/Indigenous/First Nation/Alaska Native	3	1.85%	2	3.13%	5	2.21%
Asian	13	8.02%	6	9.38%	19	8.41%
Black/African American/Afro-Caribbean/African	36	22.22%	8	12.50%	44	19.47%
Middle Eastern or North African	2	1.23%	0	0.00%	2	0.88%
Native Hawaiian or Other Pacific Islander	2	1.23%	0	0.00%	2	0.88%
White	118	72.84%	52	81.25%	170	75.22%
Other Race	2	1.23%	1	1.56%	3	1.33%
Ethnicity						
Hispanic/Latine	17	10.49%	6	9.38%	23	10.18%
Non-Hispanic/Latine	142	87.65%	58	90.63%	200	88.50%
Not specified	3	1.85%	0	0.00%	3	1.33%
Parent/Caregiver Relationship						
Mother	122	75.31%	50	78.13	172	76.11%
Father	32	19.75%	13	20.31%	45	19.91%
Relative/Other (e.g., Stepmother, Grandmother)	6	3.70%	1	1.56%	7	3.10%
Not specified	2	1.23%	0	0.00%	2	0.88%

^a^*M* = 100, *SD* = 15; ^b^*M *= 50, *SD* = 10; ^c^Race percentages exceed 100% due to participants selecting multiple racial identities; *SRS-2*  Social Responsiveness Scale, Second Edition

For the school-based study, participants demonstrated cognitive and behavioral capacity to understand the treatment material as reported by their teacher, using prompts with pragmatic language that aligns with the verbal demands of the curriculum. Participants also needed evidence of autism supported by at least one of the following: (1) eligible for and/or receiving school-based services or supports for autism, (2) prior clinical diagnosis of autism from a qualified health professional, and/or (3) characteristics of autism consistent with DSM-5 criteria, as judged by a clinical autism expert, based on all available information gathered through the screening, consenting, and testing process, including the SCQ and Social Responsiveness Scale – Second Edition (SRS-2 [28]).

Participant age did not differ significantly between the laboratory- (*M* = 15.94, range = 14.08–18.15 years) and school-based (*M* = 16.25, range = 14.14–22.15 years) study samples (*p* = .095), although the age range was broader (extending older) in the school-based sample. Study samples also did not differ significantly on autism characteristics on the SRS-2 (*p* = .338), but the range of SRS-2 scores in the high school sample (*M* = 71.77, *SD* = 11.76, range = 42–98) was wider, extending to include more individuals with lower levels of autism characteristics, than the laboratory-based sample (*M* = 73.28, *SD* = 9.91, range = 50–99). The laboratory-based sample did have a higher FSIQ on average (*M* = 109.66, *SD* = 13.13, range = 83–142) than the school-based sample (*M* = 98.06, *SD* = 16.44, range = 61–135; *t*(143.91) = -5.55, *p* < .001).

### Procedures

This study utilizes baseline data from teens who participated in two larger EF treatment studies from 2021 to 2025. At the time of enrollment, participants completed IQ testing; if records of an IQ test (e.g., from school or clinical assessment) within the previous two years were available and shared with the study team by the family, the full-scale IQ score from that report was used in lieu of repeating IQ testing. Teens and parents completed a battery of rating scales and task-based measures that was nearly identical across the two studies, spanning domains of adaptive behavior, EF, mental health, self-determination, quality of life, and social communication. Participants were provided the option to have reasonable accommodations to respond to questionnaires, including having questionnaires read to them by a member of the study team, if desired. A study team member was present during teens’ completion of questionnaires to answer any questions or provide clarification about the content of questionnaires. Participants were encouraged to inform the staff member if they needed to take a break, or if they needed any additional supports or accommodations (e.g., writing their responses). For the current paper, data from teen- and parent-report rating scales of EF and self-determination were pooled across the two studies. All study procedures were conducted in accordance with Children’s National’s Institutional Review Board. Prior to participation, informed consent was obtained from all participants 18 years of age and older and from the parent or legal guardian of all participants under the age of 18; assent was also obtained from all teens under 18 years of age. Data from the school-based trial participants were only included if consent was provided to store their de-identified study data with other research studies at our center.

### Measures

The *Behavior Rating Inventory of Executive Function*,* Second Edition (BRIEF-2; Gioia et al.*,* 2015)* is a widely used rating scale of EF in youth ages 5–18 years. Each item is rated on a three-point Likert style scale, ranging from *“Never”* to *“Often*.” The BRIEF-2 Parent Form includes 63 items that comprise nine subscales: Inhibit, Self-monitor, Shift, Emotional Control, Initiate, Working Memory, Plan/Organize, Task-Monitor, and Organization of Materials. The BRIEF-2 Self-Report Form, indicated for individuals ages 11–18 years old, includes 55 items that assess seven subscales: Inhibit, Self-Monitor, Shift, Emotional Control, Task-Completion, Working Memory, and Plan/Organize. Higher subscale T-scores (*M* = 50, *SD* = 10) indicate greater challenges within a given domain of EF, with scores of 65 or higher indicating potential concern, and scores of 70 and higher being considered clinically elevated. Although the BRIEF-2 is normed on neurotypical youth, the assessment manual contains clinical samples for reference. Research demonstrates that autistic youths’ self-report of EF challenges on this measure shows moderate concordance with parent-reported EF difficulties, supports a distinct profile from youth with ADHD and neurotypical youth, and adds additional insight and value beyond parent-report by highlighting particular areas of EF that autistic youth themselves find most challenging [13]. To allow for comparison within our study and to provide a developmentally-appropriate metric of EF, the BRIEF-2 was still used for the small subset of high school students ages 19–22 (*n* = 4), but was scored according to 18-year-old norms. For gender diverse participants, both male and female sex-normed T-scores were calculated and then arithmetically averaged.

The *American Institutes of Research (AIR) Self-Determination Scale* [29] is an informant-report measure developed to evaluate self-determination, with both a Student Form and a Parent Form available. The Student Form is a self-report version with 24 Likert-style questions anchored from *1 (“Never”)* to *5 (“Always”).* Questions are divided into four 6-item subscales: (1) “Things I Do,” (2) “How I Feel,” (3) “What Happens at Home,” and (4) “What Happens at School,” each with a total possible raw score from 6 to 30. The “Things I Do” subscale includes items such as, *“I set goals to get what I want or need. I think about what I am good at when I do this”* and *“I begin working on my plans to meet my goals as soon as possible.”* The “How I Feel” subscale includes items such as, *“I feel good about what I like*,* what I want*,* and what I need to do”* and *“I am willing to try another way if it helps me to meet my goals.”* The “What Happens at Home” subscale includes items such as, *“People at home let me know that I can set my own goals to get what I want or need”* or *“At home*,* I have learned how to make plans to meet my goals and to feel good about them.”* Finally, the “What Happens at School” subscale includes items such as, *“At school*,* I am allowed to act on my plans right away”* and *“I have someone at school who can tell me if I am meeting my goals.”* The Student Form yields a Capacity (sum of “Things I Do” and “How I Feel;” possible range = 12–60) and Opportunity (sum of “What Happens at Home” and “What Happens at School;” range = 12–60) score. The Parent Form has 18 questions that mirror the language of items on the Student Form (e.g., *“My child sets goals to get what he or she wants or needs…”*), but omits report about their child’s feelings. This results in three subscales – one related to capacity (“Things My Child Does”) and the same two opportunity-related subscales as the Student Form. For comparability across raters, the current study reports on “Capacity” (sum of “Things I Do” and “How I Feel” on the Student Form, or the single subscale “Things my Child Does” on the Parent Form) and “Opportunity at Home” (“What Happens at Home” subscale on either form). Split-half internal consistency and test-retest reliability for the AIR Self-Determination Scale is reported as 0.95 and 0.74, respectively [29]. Autistic individuals’ self-report on the AIR Self-Determination Scale has demonstrated acceptable validity and reliability, in both English and Spanish [30, 31]. 

### Analytic plan

To accomplish the first aim of evaluating the relationship between EF subdomains and self-determination within rater, four generalized linear regression models were run, as follows. First, all seven BRIEF-2 self-report subscales were entered into a model predicting (1) self-report of the AIR Self-Determination Capacity Score, and then in a separate model as predictors of (2) self-reported AIR Self-Determination Opportunity at home. Two identical models were then run within parent-report, regressing (3) parent-reported AIR-Self Determination Capacity and then (4) parent-reported AIR Self-Determination Opportunity at home, onto all nine BRIEF-2 parent-report subscale scores. For each model, regressions were run in RStudio [32] using *glmnet* [33, 34]. Alpha values were preset as described below, cross-validated to determine the optimal lambda value, and then coefficients at the optimal lambda value were extracted and reported. Elastic Net [35] balances the penalties of two common regularization techniques: (1) Least Absolute Shrinkage and Selection Operator (LASSO) and (2) Ridge. LASSO [36] regularization applies the “L1” penalty to reduce overfitting; LASSO aims to reduce several coefficients to zero in order to maintain fewer predictors with the most robust coefficients, often resulting in a more parsimonious model. In *glmnet*, this can be accomplished by increasing the alpha (*α*) parameter, commonly set to 1 to drive some predictors to coefficients of zero, and tuning the lambda (*λ*), or strength of regularization, through cross-validation to minimize shrinkage of the most important remaining predictor variables’ coefficients. By contrast, Ridge regression [37] applies the “L2” penalty to reduce large coefficients while attempting to keep all predictor variables in the model; for Ridge regression, the alpha parameter is minimized (*α* = 0), and the lambda parameter can then be tuned via cross-validation to determine the optimal amount of shrinkage. This method aims to maintain most predictors in the model, with the assumption that they are all important to a degree, but reduces overfitting by forcing lower and more evenly distributed coefficient magnitudes across the many predictors. The Elastic Net approach [35] balances L1 and L2 penalties, making it particularly effective at handling multicollinearity in models where the predictors are correlated, such as subdomains of the BRIEF-2. In the current study, we set alpha for Elastic Net models to 0.5 (i.e., the midpoint between LASSO and RIDGE parameters) and report coefficients at the optimal lambda parameter for each model. For robustness, analyses were also repeated with LASSO regularization, but the overall pattern of results was similar. The few coefficients that were forced to zero in the LASSO models but maintained in the Elastic Net models were < 0.005; in other words, when rounded to two decimal places (as in Table 2), the coefficients are equal to 0.00. As such, for parsimony and clarity, only Elastic Net results are reported; results < 0.005 (i.e., rounding down to zero) that are not stable across both Elastic Net and LASSO are indicated as such (see Table 2) and may not be as robust as predictors that survive across the two models’ parameters.

Exploratory analyses included a parallel set of four Elastic Net generalized linear regression models, but instead of evaluating the relationships between EF and self-determination *within* each rater, these relationships were evaluated *across* raters. Each of the two parent-reported subdomains of self-determination were regressed onto the self-reported EF subdomains, and then each of the two self-reported subdomains of self-determination were regressed onto the parent-reported EF subdomains.

## Results

### Within-rater analyses

Regression results are reported within rater, first within self-report and then within parent-report (see Table 2 for complete regression results). For the model with self-reported EF predicting self-reported self-determination capacity, Elastic Net determined optimal lambda parameter (*λ* = 0.47). At this parameter, self-reported EF challenges predicted self-reported capacity for self-determination (*F*(4,214) = 12.69, *R*^*2*^ = 0.192, *p* < .001); more challenges with flexibility or shifting (Shift Scale; *ß* = -0.13) and planning and organizing (*ß* = -0.22) were related to less self-perceived capacity for self-determination, as were emotion control (*ß* = -0.002) and task-completion (*ß *= -0.001), but these coefficients were extremely small and these variables were not stable in the LASSO model, suggesting they may not be as robust of predictors as shifting and planning/organizing. More EF challenges by self-report also predicted fewer self-reported opportunities for self-determination at home (*F*(5,213) = 3.50, *R*^*2*^ = 0.076, *p* = .005, *λ* = 0.40). However, five of seven EF subdomains (i.e., self-monitoring, shifting, emotion control, working memory, and planning/organizing) survived penalties with fairly small coefficients (*ß*s = -0.01 to -0.04), suggesting that this relationship may be less domain-specific within self-report, reflecting the importance of global EF for self-reported opportunities for self-determination.

Within parent-report, greater EF challenges in five of the nine subdomains were related to lower parent ratings of their teen’s self-determination capacity (*F*(5,210) = 23.76, *R*^*2*^ = 0.361, *p* < .001, *λ* = 0.36) – self-monitoring (*ß *= -0.04), flexibility (*ß *= -0.03), initiation (*ß *= -0.13), working memory (*ß *= -0.01), and planning/organizing (*ß *= -0.07). More EF challenges by parent-report were also related to fewer parent-reported opportunities for their child to demonstrate self-determination at home (*F*(5,209) = 6.68, *R*^*2*^ = 0.138, *p* < .001) at optimal lambda parameter (*λ* = 0.32), with five of nine BRIEF-2 subscales surviving penalties in the Elastic Net model (i.e., inhibition, emotion control, initiation, working memory, organization of materials) with small coefficients (0 > *ß*s > -0.03), although the effect of emotion control was negligible (*ß* = -0.002) and was not stable across models, being reduced to zero when using LASSO parameters.

**Table 2:** Elastic net results: EF sub-domains regressed onto self-determination capacity and opportunity (within- and across- rater)

	Self-Determination (Self-Report)		Self-Determination (Parent-Report)	
	Capacity	Opportunity	Capacity	Opportunity
**Executive Function (Self-Report)**	***R*** ^***2***^ = 0.192***	***R*** ^***2***^ = 0.076**	***R*** ^***2***^ = 0.028*	***R*** ^***2***^ = 0.052*
	*λ* = 0.471	*λ* = 0.404	*λ* = 0.502	*λ* = 0.125
	***ß***	***ß***	***ß***	***ß***
Inhibit	.	.	.	*-0.00*
Self-Monitor	.	-0.04	.	*0.00*
Shift	-0.13	-0.01	.	.
Emotion Control +	*-0.00*	-0.01	-0.04	.
Task-Completion +	*-0.00*	.	.	0.06
Working Memory +	.	-0.02	.	0.01
Plan/Organize +	-0.22	-0.04	*-0.00*	-0.09
**Executive Function (Parent-Report)**	***R*** ^***2***^ = 0.033*	***R*** ^***2***^ ** = 0.023** ^**†**^	***R*** ^***2***^ = 0.361***	***R*** ^***2***^ = 0.138***
	*λ* = 1.032	*λ* = 0.750	*λ* = 0.359	*λ* = 0.320
	***ß***	***ß***	***ß***	***ß***
Inhibit	.	.	.	-0.03
Self-Monitor	.	.	-0.04	.
Shift +	-0.07	-0.01	-0.03	.
Emotion Control +	.	-0.03	.	*-0.00*
Initiate	.	.	-0.13	-0.03
Working Memory +	-0.04	.	-0.01	-0.02
Plan/Organize	.	.	-0.07	.
Task-Monitor	.	.	.	.
Organization of Materials	.	.	.	-0.03

Note. ***p < .001, **p < .01, *p < .05, ^†^*p* < .1; alpha (*α*) = 0.5 for all models; lambda (*λ*) values indicate optimal regularization parameter that yields the minimum mean error through cross-validation for each generalized linear regression model. Italicized coefficients are ones that were reduced to zero under LASSO parameters. In the first column, “+” following a variable name indicates that it is a cross-rater predictor, predicting at least one self-determination variable by the opposite reporter

### Exploratory across-rater analyses

Parent-report of teens’ EF was significantly related to teens’ report of their own self-determination capacity, *F*(2,212) = 3.60, *R*^*2*^ = 0.033, *p* = .029; at tuned parameter (*λ* = 1.03), parents rating greater difficulties with their teens’ flexibility/shifting (*ß *= -0.07) and working memory (*ß* = -0.04) was related to teens self-reporting lower self-determination capacity. The model including parent-reported EF difficulties and teens’ self-reported opportunities for self-determination at home was trending toward significance (*F*(2,212) = 2.50, *R*^*2*^ = 0.023, *p* = .084), with flexibility (*ß* = -0.01) and emotion control (*ß* = -0.03) maintained in the model (*λ* = 0.75). 

Greater self-reported difficulty with emotional control (*ß* = -0.04) was related to lower capacity for self-determination by parent-report (*F*(2,216) = 3.13, *R*^*2*^ = 0.028, *p* = .046) at optimal regularization parameter (*λ* = 0.50). Self-reported planning/organization was also maintained in the Elastic Net model (*ß *= -0.002) but was not stable across models in LASSO parameters. Finally, more self-reported challenges with planning and organizing (*ß* = -0.09) related to fewer parent-reported opportunities for self-determination at home, whereas more self-reported challenges with task-completion (*ß *= 0.06) and working memory (*ß *= 0.01) actually predicted more opportunities for teens to be self-determined at home by parent-report (*F*(5,212) = 2.34, *R*^*2*^ = 0.052, *p* = .043, *λ* =  0.12). Self-report of self-monitoring (*ß *= -0.002) and inhibition (*ß* = 0.001) were also maintained in the Elastic Net model but were not stable across parameters, being reduced to zero in the LASSO model.

## Discussion

The current study is the first to evaluate the relationships between individual domains of real-world EF and self-determination in autistic teens, and to explore these domain-specific relations both within and across teen and parent reporters. We replicated previous work demonstrating that EF and self-determination are related in autistic youth and extended this work by showing that: (1) specific subdomains of EF predict self-determination, and (2) there are significant relationships between EF and self-determination when looking across, as well as within, reporters. Together, these findings elevate the utility of targeted EF intervention in building self-determination skills, which are essential for positive adult outcomes.

Specifically, we found that within self-report, more difficulty with flexibility and planning/organizing predicted lower self-determination capacity; however, the link between self-reported EF and self-reported opportunities for self-determination at home appeared to be less domain-specific in this analysis, highlighting a range of EF subdomains (e.g., self-monitor, shift, emotion control, working memory, plan/organize) as potentially important predictors of the self-determination opportunities they are provided at home. Of note, planning and flexibility have significant cognitive components that are more internalized and, therefore, less “visible” domains of EF. Teens’ ability to self-report more fully on these components beyond what their parents can readily observe may allow them to point more specifically to challenges in these domains as interfering with their own perceived capacity for self-determination.

Within parent report, several subdomains of EF were related to self-determination capacity and opportunity, which may suggest that parents’ perception of their child’s self-determination and the opportunities they provide their child to be self-determined may be influenced by EF more broadly, or in a less domain-specific way. However, only EF subdomains of initiation and working memory spanned both capacity and opportunity within parent-report, whereas other EF domains were differentially related to capacity or opportunity. Namely, self-monitoring, flexibility, and planning appeared important for parents’ perception of their teen’s capacity for self-determination, which aligns quite well with the relationships seen within our teens’ self-report. On the other hand, parents’ view of their teen’s inhibition and organization of materials, which are highly visible EF domains, appeared relatively more important for the opportunities they provide for their child to be self-determined at home.

In exploratory analyses across rater, more parent-reported EF challenges in flexibility and working memory predicted lower self-reported capacity for self-determination, whereas greater parent-reported problems with emotion regulation and flexibility related to teens perceiving that they have fewer opportunities for self-determination at home. More self-reported emotional control difficulties were related to worse parent-reported self-determination capacity. Finally, as expected, more self-reported EF difficulties with planning and organizing predicted fewer parent-reported opportunities. Surprisingly, however, more self-reported difficulties with task completion and working memory were related to more opportunities for self-determined behavior by parent-report, the only positive associations between EF difficulties and self-determination in our analyses.

### Interpretation of findings within extant literature

Our findings replicate and extend those of limited prior literature with autistic teens suggesting relationships between overall EF ratings and self-determination capacity within rater [21], by highlighting potential domain-specific EF skills that may relate to self-determination capacity and opportunity, as well as cross-rater links between EF and self-determination. In fact, several domain-specific relationships emerged across raters, whereas some within-rater relationships suggested the importance of EF more globally. The broader literature finds similar concerns with rater bias when using within-rater comparisons, as shared method variance is often captured over individual constructs [38]. In the current study, we found that greater difficulty with flexibility (or shifting) significantly predicted lower self-determination capacity and/or opportunity within self-report, within-parent report, and in some cross-rater analyses, suggesting that flexibility may be a particularly central EF subdomain in relation to self-determination that is robust across raters. This makes sense given that flexibility is a central tenet of self-determination frameworks [1], as it is necessary to shift to a new approach or plan if one’s initial plan is not working well or an obstacle is encountered, or to return one’s attention back to goal-oriented tasks after interruptions. Additionally, as predicted, planning also predicted self-determination capacity and/or opportunities within and across reporters. This aligns with Lui and colleagues’ [24] finding that task-based planning was related to self-determination in adolescents/young adults with intellectual disability, where approximately half of their sample had an autism diagnosis, suggesting that planning may be an EF skill that relates to self-determination across intellectual ability levels. This is important within the context of prior work finding that teens and parents rated items on the AIR Self-Determination Scale related to planning and monitoring of plans as the lowest opportunities autistic teens receive to be self-determined in the home environment [15].

Within parent-report, results pointed to the importance of EF broadly. However, better initiation was significantly related to both higher perceived teen capacity for self-determined behavior, as well as more parent-reported opportunities for their teen to be self-determined at home. It makes sense at face value that taking initiative to pursue goals and related steps would relate to agency in plans and goals. In fact, some self-determination theorists consider initiative to be a related but distinct concept from autonomous action in self-determination, where initiative requires more internal motivation, as well as structuring of activities so that they are sufficiently challenging and/or enjoyable for the individual [39, 40]. To this end, parents may be less likely to view teens as capable of initiating and executing their goals on their own when they perceive them to lack initiative or motivation to do so, and may in turn be less likely to allow their teen independence to create and follow through on their own goals. Of note, initiation is not a skill assessed on the self-report form of the BRIEF-2. Additionally, parent-reported disinhibition in their teens was uniquely related to fewer parent-reported opportunities for self-determination. This finding aligns with papers on self-determination theory [1] that highlight inhibitory control as a core facilitators of agentic action.

Emotional control demonstrated within- and cross-rater relationships with self-determination, including for predicting opportunity in the current study. It is likely that, in the presence of great emotion regulation difficulties, parents are more hesitant to provide opportunities for independent self-determination that may trigger frustration or meltdowns. Working memory also demonstrated some cross-rater, as well as within rater, relationships with self-determination. This may underscore the importance of supporting teens’ working memory, such as by teaching them working memory strategies, as difficulty with working memory has clear downstream effects on the ability to carry out plans and achieve goals. Finally, our finding that greater self-reported challenges with task-completion and working memory related to more parent-reported opportunities for self-determination at home was surprising. If replicated in future studies, research should seek to better understand this unexpected relationship. For example, it is possible that a teen viewing that they have more difficulty completing tasks or with working memory makes parents likely to provide more scaffolded opportunities for their child to engage in self-determined planning and goal setting, in hopes of facilitating better task completion. On the other hand, when parents provide more opportunities to independently complete tasks, teens may become more self-aware of their difficulties completing tasks and independently employing working memory. It is also possible that parents and teens are simply experiencing and reporting on various aspects of EF and perceived opportunities for self-determination differently from each other.

### Clinical implications

If replicated, these findings have significant implications for supporting autistic teens’ self-determination. In particular, it may be especially important to provide intervention or support for building specific EF skills such as planning in pursuit of teens’ goals, initiative to start or continue executing these plans, flexibility in the event of a plan not working, and inhibition and emotional control in the event of distractions or other external events that may derail their plans. Further, opportunities are known to be essential in order to practice and develop skills, but are also crucial for individuals to have the chance to demonstrate to themselves and to others (e.g., parents/caregivers, teachers) that they can use these skills successfully [41]. Such opportunities allow for teens themselves and the adults around them to update their perceptions of teens’ capacity to act in a self-determined way and, in turn, provide more opportunities in the future, rather than assuming limited capacity based on history or never having attempted the skills independently. This might include providing increased opportunities for teens to engage in goal-setting, developing plans to meet those goals, and initiating steps toward their own plans and goals, with diminishing guidance from parents/caregivers over time. Additionally, supporting teens to learn and practice how to effectively self-advocate, both for the opportunities they want and for any accommodations or supports they may need (e.g., working memory supports), can increase their success in actuating these skills and enhance overall self-determination and autonomy.

It should be noted that the current study was described to families specifically as an intervention focused on helping with preparation for post-secondary education through skills such as goal setting, flexibility, planning, self-advocating, and motivation. As such, it is likely that the participant sample skews toward representing autistic individuals with relatively higher EF needs. However, EF challenges are highly prevalent in autism [42], making them a crucial treatment target to study in research trials such as this one.

### Limitations and future directions

The current study is limited by the exploratory nature of our analyses, particularly for cross-rater aims, due to limited prior research on these constructs. However, the Elastic Net approach was deliberately chosen to avoid overfitting our models and to limit concerns of multicollinearity among BRIEF-2 subscales. Further, the current study utilized only rating scale measures of EF and self-determination. This is an intentional decision to capture real-world EF, as many task-based measures lack ecological validity [19], as well as to prioritize self-report of EF and self-determination, which are not centered in many studies. However, in their conceptual review of assessment and treatment for autistic adolescents and young adults, Shogren and colleagues [17] recommend that clinical care might be best informed by incorporating self-report of self-determination and objective (i.e., task-based) measures of EF. Future research may do well to incorporate these two methods of measurement, but with a particular focus on ecologically-valid EF tasks that involve executing everyday tasks that rely on EF skills (e.g., making plans for real-world scenarios, and generating flexible alternatives if those initial plans are disrupted). Additionally, the current study looks only at concurrent relationships between EF and self-determination, which precludes making any causal claims about the variables. Future research should evaluate these relationships longitudinally and/or in response to EF intervention, to determine whether improving EF leads to improvements in self-determination, and whether improved self-determination may mediate relationships between EF changes and improvements in more distal real-world outcomes of importance to autistic people (e.g., adaptive behavior, mental health). The current study sample is relatively limited in terms of racial, ethnic, and gender diversity. Future research should seek to replicate these results in a more diverse sample to evaluate how these intersecting identities may impact the relationship between EF and self-determination. Given that research focused on self-determination is still in relatively early stages, future studies should seek to evaluate additional predictors of self-determination, such as whether age, gender, or other diagnoses (e.g., ADHD) impact capacity and opportunities for self-determination, or whether these factors moderate the relationship between EF and self-determination.

### Conclusions

The current study is the first to evaluate specific domains of real-world EF in relation to self-determination capacity and opportunity in autistic teens, and the first to do so across parent- and self-report. Findings highlight skills such as planning and flexibility as specifically salient within self-report of capacity for self determination, whereas a broader range of skills (including flexibility and planning) appeared to be important within parent-report, suggesting potentially more global relationships. Flexibility, planning, working memory, and emotion regulation were particular subdomains of EF that demonstrated cross-rater relationships with self-determination. Further, skills such as emotional control, organization of materials, and inhibition may be particularly important for understanding the opportunities provided for teens to be self-determined. Future studies should explore these relationships longitudinally and with multi-modal assessment measures to increase robustness. Nonetheless, initial findings suggest the importance of supporting specific EF skills (e.g., planning, flexibility, inhibition, initiation, emotional control) that may be most important for facilitating self-determination, as well as the importance of providing more opportunities for autistic adolescents to exhibit self-determined planning and goal-setting in pursuit of their goals.

## Data Availability

The datasets analyzed during in the current paper are available from the corresponding author upon reasonable request.
